# Maxent modeling for predicting the spatial distribution of three raptors in the Sanjiangyuan National Park, China

**DOI:** 10.1002/ece3.5243

**Published:** 2019-05-20

**Authors:** Jingjie Zhang, Feng Jiang, Guangying Li, Wen Qin, Shengqing Li, Hongmei Gao, Zhenyuan Cai, Gonghua Lin, Tongzuo Zhang

**Affiliations:** ^1^ Key Laboratory of Adaptation and Evolution of Plateau Biota, Northwest Institute of Plateau Biology Chinese Academy of Sciences Xining China; ^2^ University of Chinese Academy of Sciences Beijing China; ^3^ Qinghai Provincial Key Laboratory of Animal Ecological Genomics Xining China; ^4^ Qinghai Provincial Environmental Protection Department Xining China; ^5^ Qinghai Academy of Animal Science and Veterinary Medicine Qinghai University Xining China

**Keywords:** habitat suitability, Himalayan Vulture (*Gyps himalayensis*), Maxent, Saker falcon (*Falco cherrug*), Sanjiangyuan National Park, Upland buzzard (*Buteo hemilasius*)

## Abstract

Upland buzzard (*Buteo hemilasius*), Saker falcon (*Falco cherrug*), and Himalayan vulture (*Gyps himalayensis*) are three common large raptors in the Sanjiangyuan National Park (SNP), China's first national park. Among them, Upland buzzard and Saker falcon play a significant role in controlling plateau rodent populations and reducing the transmission of pathogens carried by rodents. The Himalayan vulture can provide services for the redistribution and recycling of nutrients in the ecosystem, and play an irreplaceable role in the celestial burial culture of Tibetans in China. Exploring their habitat suitability is important for the protection of the three raptors. Our research was based on the current distribution of Upland buzzard, Saker falcon, and Himalayan vulture that we had extensively surveyed in the Sanjiangyuan National Park from 2016 to 2017. Combined with the correlation analysis of environmental variables, we utilized maximum entropy model (MaxEnt) to evaluate and compare the habitat suitability of the three species in the Sanjiangyuan National Park. Elevation, climate, and human disturbance factors, which had direct or indirect effects on species survival and reproduction, were all included in the model. Among them, elevation was the most important environmental variables affecting the suitability of habitats of three species. Temperature‐related factor was another important predictor. The high (>60%) suitable habitat areas for Upland buzzard, Saker falcon, and Himalayan vulture were 73,017.63, 40,732.78, and 61,654.33 km^2^, respectively, accounted for 59.32%, 33.09%, and 50.08% of the Sanjiangyuan National Park and their total suitable area (i.e., the sum area of high and moderate habitats) reached 96.07%, 60.59%, and 93.70%, respectively. Besides, the three species have overlapping areas for the suitable habitats, which means that overlapping areas should be highly valued and protected. Therefore, understanding the distribution of suitable habitats of the three raptors can provide useful information and reasonable reference for us to put forward suggestions for their protection and regional management.

## 1 INTRODUCTION

Habitat is an important place for the survival, reproduction, and population development of organisms. Its quality can directly affect the distribution, quantity, and survival rate of organisms (Block & Brennan, 1993; Hall, Krausman, & Morrison, 1997). At present, habitat loss or fragmentation is one of the most important factors threatening the survival of organisms (Brooks et al., 2002; Haddad et al., 2015; Sony, Sen, Kumar, Sen, & Jayahari, 2018), and understanding the habitat suitability and influencing factors of species habitat is fundamental to protect threatened species (Austin, 2002; Margules & Pressey, 2000). From an ecological point of view, environmental predictors can limit distribution of organisms and affect their habitat suitability (Sexton, McIntyre, Angert, & Rice, 2009; Wiens, 2011). Ecological niche models can be considered as excellent tools for predicting the habitat suitability, potential distribution of species, and the importance of environmental predictors by using distribution points and environmental variables (Mota‐Vargas, Rojas‐Soto, Lara, Castillo‐Guevara, & Ballesteros‐Barrera, 2013; Peterson, Ball, & Cohoon, 2002).

Upland Buzzard *(Buteo hemilasius),* Saker falcon *(Falco cherrug),* and Himalayan Vulture (*Gyps himalayensis)* are three large raptors, which frequently require large territories such as plateaus, mountains, and grassland (Tinajero, Barragán, & Chapa‐Vargas, 2017). The Upland Buzzard was listed as least concern species by the International Union for Conservation of Nature (IUCN). It is an important protected species in China and was classified as a Category II protected species in China (Zheng et al., 2002). The Upland Buzzard is a widespread species in the world, and the Qinghai–Tibet Plateau and the Inner Mongolia Plateau are its mainly distributed area in China. The Saker falcon occurs in a wide range across the Palearctic region from Eastern Europe to Western China, and the most species are distributed in China, Kazakhstan, Mongolia, and Russia. It is the only member of its genus to be categorized as endangered class in the IUCN Red List (BirdLife International, http://www.birdlife.org). Current estimates illustrate that the overall population trend during the 19‐year period 1993–2012 equates to a 47% decline (BirdLife International, http://www.birdlife.org), and this phenomenon was primarily caused by global exports and habitat degradation (Chavko, 2010; Levin, 2011; Stretesky, McKie, Lynch, Long, & Barrett, 2018). The increasing demand for commercial development is the main reason for Saker falcon being trapped and traded, and this transaction mainly occurs between the Middle East and Asia (Dixon, 2016; Levin, 2011; Shobrak, 2015). Himalayan Vulture is the heaviest scavenging vertebrate in Asia (Sherub, Fiedler, Duriez, & Wikelski, 2017), and the species is mainly distributed in the west and central regions in China, especially in the Qinghai–Tibet Plateau. There are also some wintering populations in Yunnan. Due to the expected impacts of diclofenac use in livestock, especially in South Asia (Das, Cuthbert, Jakati, & Prakash, 2011), it is suspected its next three generations would decline by 25%–29% (Paudel et al., 2016), so the species had been up listed to “Near Threatened” by the IUCN. Also, this species is particularly vulnerable because long‐term, long‐distance migration can cause instability in its life cycle (Dodge et al., 2014; Ricklefs & Wikelski, 2002). Raptors are difficult to observe and found because many of them are in inaccessible places (e.g., on the tops of the tallest trees or on the large vertical cliffs), they also have strong mobility. So the prediction model can provide convenience and possibility for us to understand their suitable habitat distribution (Bildstein & Bird, 2007).

Raptors are generally recognized as top predators and scavengers in the ecosystem (Donázar et al., 2016; McClure et al., 2018). Upland Buzzard and Saker falcon play important roles in controlling the population of rodent species in the plateau and reducing the transmission of pathogens carried by rodents (Sekercioglu, 2006; Tinajero et al., 2017). Himalayan Vulture can provide services for nutrient redistribution and recycling of ecosystems (Devault, Rhodes, Olin, & Shivik, 2003). It also plays an irreplaceable role in the traditional sky burial culture of the Tibetan people in China (Lu, Ke, Zeng, Gong, & Ci, 2009). Some studies have demonstrated that raptors can be regarded as “indicators of biodiversity,” indicating a positive and direct relationship can be established between the raptors and the richness of other plant and animal species in the same domain (Burgas, Byholm, Parkkima, & Thompson, 2014; Martín & Ferrer, 2013; Sergio, Newton, Marchesi, & Pedrini, 2006). Since the three raptors face certain threats or a predictable decline in population, it is therefore necessary to assess the distribution of suitable habitats targeting these three raptors in China and make suggestions for their protection and management (Mateo‐Tomas & Olea, 2010). In addition, raptors as umbrella species can benefit other species when they are attached great importance and protected (Oliveira, Olmos, Santos‐Filho, & Bernardo, 2018).

Since the Sanjiangyuan National Park is located in the Qinghai–Tibet Plateau, sensitive and changeable climate and high altitude are two significant environmental variables in this region (Guo et al., 2016). The habitat suitability of animal is often related to food resources and opportunities to avoid natural enemies (Zhang & Zheng, 1999). In the Qinghai–Tibet Plateau, the elevation gradient can determine the food resources richness of the three species. In addition, climatic variables play an important role in determining species distribution (Newton, 2003b; Thapa et al., 2018; Virkkala, Luoto, Heikkinen, & Leikola, 2005), and in particular, temperature is an important factor that drives species’ distribution (Grinnell, 1917; Guisan & Zimmermann, 2000). Climate‐related factors such as temperature and precipitation can affect not only the behavior and physiology of organisms directly, but also indirectly animal food resources by affecting vegetation (Delgado, Morales, Traba, & Garcia De la Morena, 2009). A large number of studies have shown that climate change has a great impact on species diversity (Li, 2019; Subba, Sen, Ravikanth, & Nobis, 2018). Food supply is the most direct factor to determine the survival and reproduction of wildlife (White, 2008). Considering that elevation and climate are closely related to the food resources for wildlife in plateau areas, we suppose that elevation and climate are the main predictors affecting the distribution of suitable habitats. Human disturbance is also considered as an important factor affecting wildlife. At present, human activities have accelerated the global rate of biodiversity loss, leading to an extinction crisis (Dirzo & Raven, 2003). Therefore, altitude, climate, and human influence index were used to predict the spatial distribution of raptors' suitable habitats in this study. Our objectives were to (a) exploring how environmental predictors affect habitat suitability of species and the importance of these predictors, (b) evaluating the appropriate state of habitat of the three species and the overlapping areas of their suitable habitats, and (c) providing rationalization proposals for raptors protection and habitat management in the Sanjiangyuan National Park.

## 2 MATERIAL AND METHODS

### 2.1 Study area

Sanjiangyuan National Park is the first National Park in China，which is located in hinterland of the Qinghai–Tibet Plateau, China (32°22'‐36°47'N and 89°50'‐99°14'E). The area covers 123,100 km^2^ and accounting for 31.16% of the entire area of the Sanjiangyuan. It consists of three regions: the Yangtze River Source, the Yellow River Source, and the Lancang River Source. At present, it is not only an important freshwater supply site for China and Southeast Asia (Liu, Li, & Wen, 2006; Wang, Song, & Hu, 2010), but also one of the most sensitive areas for global climate change response (Liu et al., 2017). The park is one of the regions with the highest altitude, the largest area, and the most concentrated distribution in the world (Fan et al., 2010). It is a typical plateau continental climate, mainly for the alternation of cold and hot seasons, dry and wet seasons (Yi, Li, & Yin, 2013). The rich flora and fauna in this region make it one of the most biologically diverse in the world. Acting as the first system pilot in China, the Sanjiangyuan National Park will be turned into both the exhibition of nature conservation and a heritage area of ecological culture on the Qinghai–Tibetan Plateau. Its ecosystem service function, natural landscape, and biodiversity have national and global significance (Qiao et al., 2018). The Sanjiangyuan National Park is a biodiversity hotspot area in Western China, which supports many endangered and endemic wildlife. Therefore, it is considered as the ecological security barrier of China.

### 2.2 Modeling approach

Maximum entropy model (Maxent) can use environmental variables and species presence points to calculate the constraints and explores the possible distribution of maximum entropy under this constraint condition, and then predicts the habitat suitability of the species at the study area (Merow, Smith, & Silander, 2013; Phillips, Anderson, & Schapire, 2006). Maxent is very prevalent in niche modeling because it only needs presence points and more accurate predictions can be obtained even with small sample sizes (Pearson, Raxworthy, Nakamura, & Townsend Peterson, 2006; Phillips & Dudík, 2008; Saupe et al., 2015). In the Phillips’ study, maximum entropy model was found to be the best in both predictive performance and model stability while compared with other similar niche models (Barker et al., 2006; Phillips et al., 2006).

### 2.3 Occurrence data

During July and August 2016 and 2017, we conducted a large number of field investigations in the Sanjiangyuan National Park and recorded the occurrence records of wildlife in the field survey with GPS.

**Figure 1:**
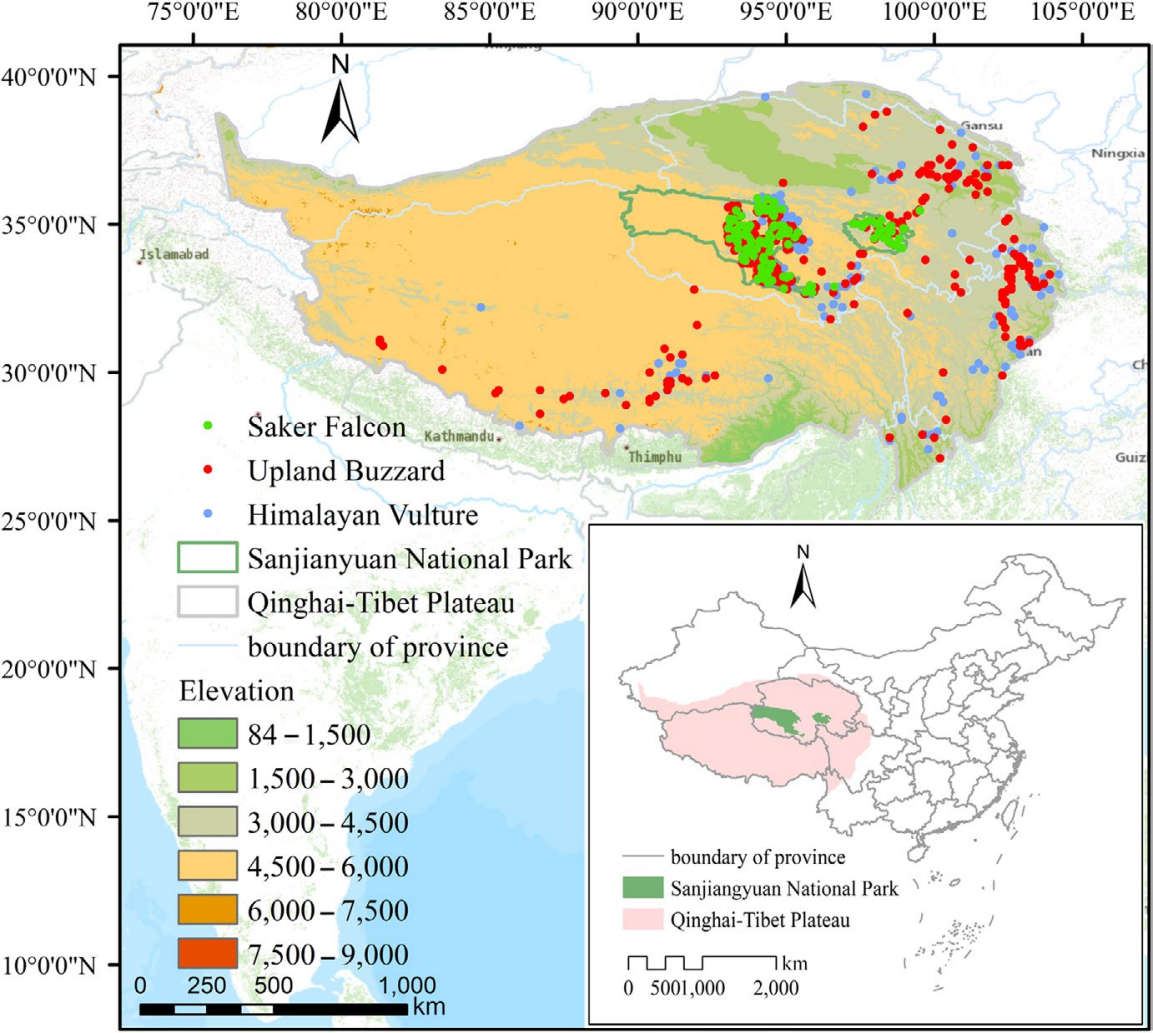
The presence points of Upland Buzzard*,* Saker falcon, and Himalayan Vulture on the Qinghai–Tibet Plateau

The presence data of Upland Buzzard, Saker falcons, and Himalayan Vulture were collected from Global Biodiversity Information Facility (GBIF) (https://www.gbif.org/) and wildlife resources data in the Sanjiangyuan National Park surveyed by the Northwest Plateau Institute of Biology, Chinese Academy of Sciences in 2015–2017. In total, we collected 642, 162, and 366 effective GPS sites for Upland Buzzard*,* Saker falcon, and Himalayan Vulture. Among them, 426, 157, and 158 presence points of the Upland Buzzard*,* Saker falcon, and Himalayan Vulture were obtained respectively from our field surveys, and all others were from GBIF website. The accuracy of the model would be affected by spatial autocorrelation if the distance between sample points is too close, so we randomly removed a point with distances less than 1 km between two points of the three species (Milchev, 2009). In addition, we used the elevation of three species' resting sites, nesting sites, and feeding sites to represent the elevation gradient of the three species. Finally, 592, 153, and 356 presence points of Upland Buzzard*,* Saker falcon, and Himalayan Vulture were reserved for model operation (Figure 1).

### 2.4 Environmental variables and processing

The selection of environmental variables mainly considers their restrictive effect on species distribution and spatial correlation among variables (Peterson et al., 2011). In this study, 67 climatic variables were obtained from WorldClim1.4 (1950–2000) (http://www.worldclim.org/), including 19 bioclimatic variables (Bio1‐Bio19) and 48 climatic variables that describe monthly total precipitation and average, minimum, and maximum monthly temperature. Climatic variables were frequently used in habitat modeling due to direct effects on species distribution (Guisan & Zimmermann, 2000). Elevation was extracted from an ASTER GDEM V2 digital elevation model (DEM; http://www.gscloud.cn/). The human influence index (HII) was obtained from Last of the Wild, v2 (http://sedac.ciesin.columbia.edu/), representing anthropogenic impacts spanning 1995–2004 that were calculated by integrating the data including human population pressure (population density), human land use (built‐up areas, nighttime lights, land use, and land cover), and human accessibility (coastlines, roads, railroads, and navigable rivers). All the above environmental variables have a high generality to the topography, climate, and human disturbance of the study area. The spatial resolution of all these environmental variables is 1 km^2^.

We used ArcGIS 10.5 to convert the three raptors distribution points (csv format) into raster data firstly. Then the attribute values of 68 environmental variables for three species were extracted using the Spatial Analyst tool in the ArcGIS toolbox. The downloaded layer format of environment variables also needed to be converted to the ASCII format required by the Maxent software. As too many environmental variables would increase the complexity of the model and the random error and reduce the accuracy of the prediction results, the SPSS22 (Statistical Product and Service Solutions) software was used to perform principal component analysis (PCA) and correlation analysis on the attribute values of 68 environmental variables of the three species (Jiang, 2018). Among the 67 climatic variables we selected, 19 bioclimatic and 48 other monthly related climatic variables have great similarities. In addition, the elevation gradient is also related to climate and human disturbance factors. Therefore, in order to avoid the high similarity among these environmental variables, we removed the factors that the Pearson's correlation coefficient is greater than or equal to 0.8 from the Pearson correlation coefficient table. Finally, we screened 11, 11, and 10 environmental variables for Upland Buzzard*,* Saker falcon, and Himalayan Vulture, and all the selected variables included climate, altitude, and human influence factors. So they can be used for subsequent modeling (Table S1).

### 2.5 Model procedure

Maxent can produce higher quality output if we optimize or adjust the model settings (Sometimes called “smoothing”) instead of the default settings to estimate the optimal model complexity (Anderson & Gonzalez, 2011; Warren & Seifert, 2011). Feature combination (FC) parameters and regularization multiplier (RM) are two important parameters. Feature combination can transform environmental variables mathematically, so that Maxent can use complex mathematical relationships to infer species' response to environmental factors. Five characteristic parameters (linear [L], quadratic [Q], hinge [H], product [P], and threshold [T]) are combined. Regularization multiplier is a new constraint added to the model on the basis of the characteristic parameters, and it adjusts the model to simulate the response curve by changing RM value. We increased this parameter from a default value of 1, by 0.5 at a time. The ENMeval data package evaluates the complexity of the model by testing the AIC values (AICc) corrected by the Maxent under different parameter conditions. Akaike information criterion correction (AIC) is a standard to measure the goodness of statistical model fitting, and it generally gives priority to the parameters with small AIC values for simulation.

In addition, we introduced the presence points of the three raptors and the selected environmental factors into the Maxent model, randomly selected 75% of the species distribution points to build the model, and the remaining 25% of the species distribution points to test the model. Jackknife tests are used to analyze the contribution rate and importance of variables, and the model accuracy can be judged as excellent if AUC value is between 0.9 and 1, good if AUC value is between 0.8 and 0.9, fair if AUC value is between 0.7 and 0.8, poor if AUC value is between 0.6 and 0.7, and failed if AUC value is between 0.5 and 0.6 (Swets, 1988). The suitability maps were calculated using the logistic output of Maxent, and the logical habitat suitability index we obtained is from the lowest "0" to the highest "1." We divided the habitat suitability maps into four grades according to the expert experience method (i.e., this classification method has been applied to a large number of studies): 0–0.2 is unsuitable; 0.2–0.4 is low; 0.4–0.6 is moderate; and 0.6–1 is high (Ansari & Ghoddousi, 2018; Convertino, Muñoz‐Carpena, Chu‐Agor, Kiker, & Linkov, 2014).

Finally, we used ArcGIS 10.5 to superimpose the suitable habitat of the three species, and the superimposed result can show the common suitable habitats of the three raptors.

## 3 RESULTS

### 3.1 Model performance

**Figure 2:**
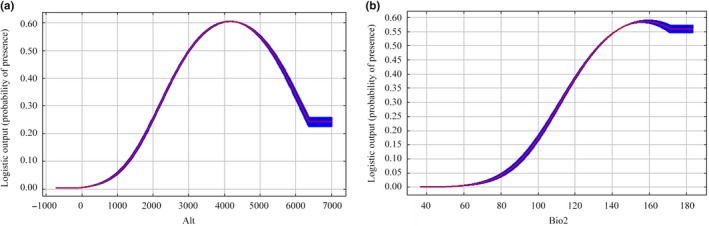
Response curves of environmental variables of Upland Buzzard: (a) Elevation (m) and (b) Mean diurnal range (°C)

When the AIC and BIC values of the three raptors were the smallest and the response curve reached the best effect, the characteristic values of the Upland Buzzard were L, Q, P, and RM = 2；The characteristic values of the Saker falcon were L, Q, P, and RM = 2; The characteristic values of Himalayan Vultures were L, Q, P, and RM = 2. The three species were predicted using Maxent, and the results showed that the average AUC of the three species obtained after 10 repetitions were 0.893, 0.975, and 0.923, respectively, (Figure S1), which was close to 0.9 or higher than 0.9 (Figure 2), indicating the prediction result is close to or above the excellent level. Therefore, the model was highly informative and could be used for subsequent research.

### 3.2 Environmental variable importance

The results of the Jackknife test indicated the factors that contributed more to the habitat suitability of Upland Buzzard were elevation (40.3%) and mean diurnal range (31.4%). The total contribution rate of these two factors reached 71.7% while total precipitation‐related factors only accounted for 10.5% and human influence index accounted for 2.8%. The main factors affecting the suitability of Saker falcon were elevation (25.7%), mean diurnal range (21.7%), lowest temperature in December (17.0%), and highest temperature in January (12.6%). The total contribution rate was 77.0% while the total contribution rate of precipitation‐related factors was only 5.0% and human influence index only accounts for 0.6%. The main factors affecting the habitat suitability of Himalayan Vulture were elevation (44.7%) and mean diurnal range (25.1%), with a total contribution rate of 69.8% but the total contribution rate of precipitation‐related factors accounted for 13.2%, human influence index accounted for 5.5%. Therefore, elevation and temperature‐related factors were the two most important factors affecting habitat suitability. Human influence and precipitation‐related factors also affected habitat suitability, but they were not important factors (Table 1).

**Table 1:** Analysis of contribution of environmental variables of the three raptors

Code	Environmental variable	Contribution		
		Buteo hemilasius	Falco cherrug	Gyps himalayensis
Alt	Elevation	40.3	25.7	44.7
Bio2	Mean diurnal range	31.4	21.7	25.1
Bio3	Isothermality (BIO2/BIO7)(*100)	2.5	5.5	3.8
Bio6	Min temperature of coldest month	‐	‐	3.0
Bio7	Temperature annual range	7.5	4.1	4.8
Bio12	Annual precipitation	‐	‐	5.5
Bio13	Precipitation of wettest month	4.1	0.3	‐
Bio14	Precipitation of driest month	‐	‐	0.1
Bio15	Precipitation seasonality	3.1	3.1	5.1
Prec1	January precipitation	1.0	‐	2.5
Prec2	February precipitation	‐	‐	‐
Prec4	April precipitation	0.8	‐	‐
Prec5	May precipitation	‐	1.6	‐
Prec7	July precipitation	1.5	‐	‐
Tmax1	January maximum temperature	5.0	12.6	‐
Tmean10	October mean temperature	‐	7.7	‐
Tmin12	December minimum temperature	‐	17.0	‐
HII	Human influence index	2.8	0.6	5.5

### 3.3 Single environmental variable analysis

**Figure 3:**
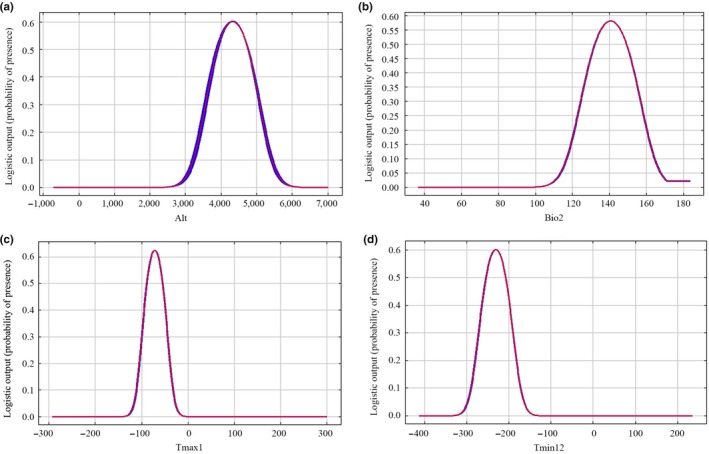
Response curves of environmental variables of Saker falcon: (a) Elevation (m), (b) Mean diurnal range (°C), (c) Maximum temperature range (°C), and (d) Minimum temperature range (°C)

**Figure 4:**
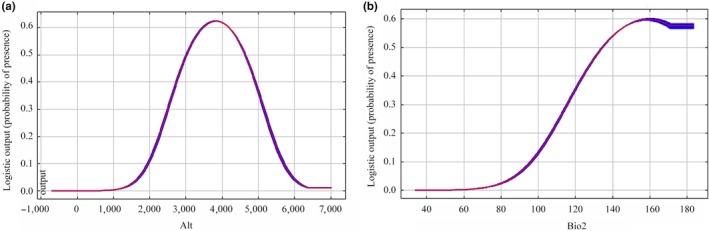
Response curves of environmental variables of Himalayan Vulture: (a) Elevation (m) and (b) Mean diurnal range (°C)

We selected environmental factors that contributed rate more than 10% to analyze single factor of three raptors (Figures 2, 3, 4; Table S2).

- We discovered the habitat suitability of Upland Buzzard was greatly affected by elevation and mean diurnal range (Bio2) according to the prediction results of the model (Figure 2). The elevation range was from 0 to 5,238 m, and the optimum range was 3,500 m–5,000 m. The mean diurnal range of Upland Buzzard was between 6.5 and 16.1°C, and the optimum range was 15–16°C.
- For Saker falcon, elevation, mean diurnal range (Bio2), the January maximum temperature (Tmax1), and December minimum temperature (Tmin12) were four main environmental factors (Figure 3). The elevation range was 2,189 m–5,040 m, and the optimal range for this species was 4,200 m–4,800 m. The mean diurnal range was 11.2–14.6°C, and the optimum range was 13–14.5°C. The maximum temperature range in January was between −15.9 and −2.2°C, and the optimum range was −10 to −5.5°C. The December minimum temperature range was −25.6 to −16.5°C, and the optimum range was −24 and −21°C.
- Elevation and mean diurnal range (Bio2) were two most important factors affected the habitat suitability of Himalayan Vulture (Figure 4). We found that elevation range was 3,500 m–5,000 m and the optimum altitude range was 3,000 m–4,500 m. The mean diurnal range was from 8.6 to 16.1°C, and the optimum range was 15 to −16°C.

### 3.4 Habitat suitability maps

**Figure 5:**
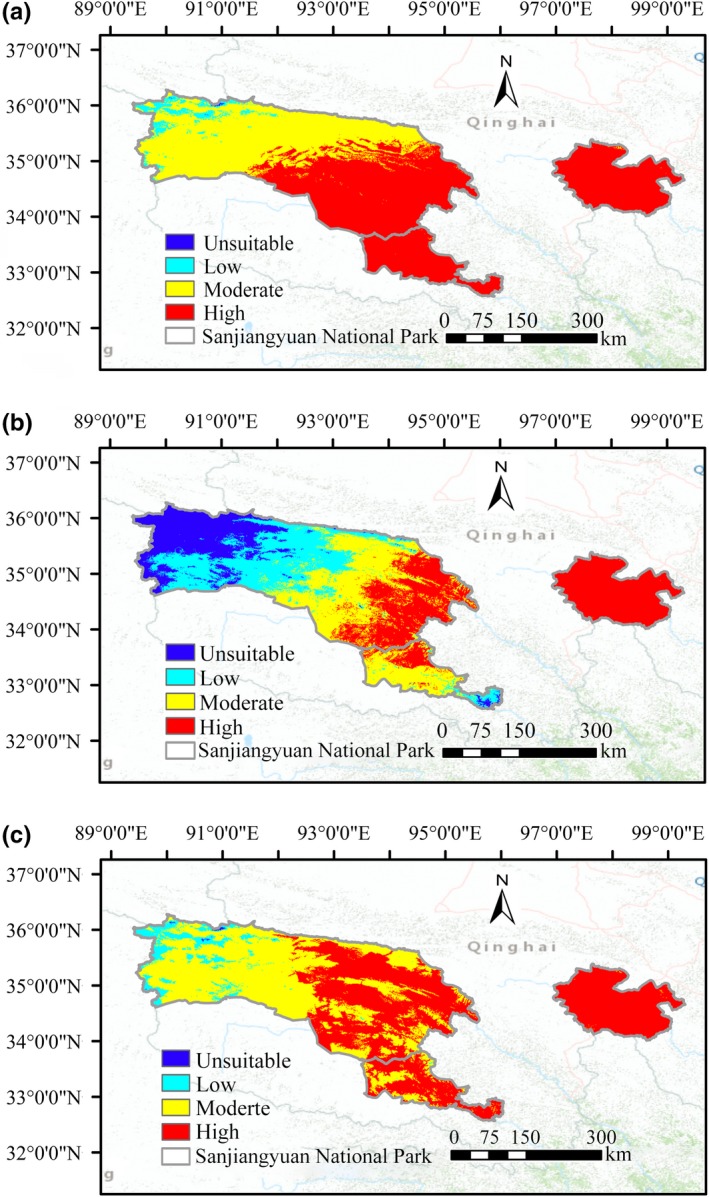
Habitat suitability maps for three raptors. (a) Upland Buzzard; (b) Saker falcon; (c) Himalayan Vulture. Different habitat suitability levels show different colors: red represents high suitability; yellow represents moderate suitability; Teal represents low suitability; and blue represents unsuitability

According to the suitability distribution maps and the suitability proportion of three raptors, we can find that the high suitable habitat area of Upland Buzzard, Saker falcon, and Himalayan Vulture were, respectively, 73,017.63 km^2^, 40,732.78 km^2^, and 61,654.33 km^2^ and they, respectively, accounted for 59.32%, 33.09%, and 50.08% of the total area of the Sanjiangyuan National Park (Table 2). For Upland Buzzard, the 99.77% area of the Lancang River source and the 99.40% Yellow River source were high‐suitability habitat, and its high suitable habitat reached 44.81% in the Yangtze River source and was mainly concentrated in southeastern parts of this area. The high suitable habitat proportions of Himalayan Vulture were high in the Lancang River source and Yellow River source, reached 72.31% and 99.46%, respectively. The high suitable habitat area was also mainly distributed in southeast of the Yangtze River source and reached 36.37%. The high‐suitability habitat of the Saker falcon also occupied almost the entire Yellow River source, with a ratio of 100%. However, the proportion of high suitable habitats of the species was very low in the Yangtze River source and the Lancang River source, accounted for only 20.85% and 20.93%, respectively (Figure 5). The overlapping suitable habitats of the three raptors were concentrated in the Yellow River source, the Lancang River source, and the southeastern part of the Yangtze River source. The total overlapping area reached 74,438.57 km^2^, accounted for 60.47% of the total area of the park (Figure S2).

**Table 2:** Habitat suitability percentage and area of three raptors

Species	Classes	Whole area		Yangtze River Source		Lancang River Sour		Yellow River Source	
		Area	Proportion (%)	Area	Proportion (%)	Area	Proportion (%)	Area	Proportion（%）
Upland Buzzard	High	73,017.63	59.32	40,462.07	44.81	13,668.87	99.77	18,986.33	99.40
	Moderate	45,239.54	36.75	45,004.84	49.84	31.13	0.23	113.67	0.60
	low	4,750.07	3.86	4,740.53	5.25	–	–	–	–
	Unsuitable	92.75	0.08	92.57	0.10	–	–	–	–
Saker falcon	High	40,732.78	33.09	18,823.88	20.85	2,867.38	20.93	19,100.00	100
	Moderate	33,849.39	27.50	25,206.63	27.91	8,651.66	63.15	–	–
	Low	27,542.72	22.37	25,761.98	28.53	1,746.47	12.75	–	–
	Unsuitable	20,975.11	17.04	20,507.51	22.71	434.49	3.17	–	–
Himalayan Vulture	High	61,654.33	50.08	32,845.29	36.37	9,905.88	72.31	18,996.21	99.46
	Moderate	53,693.56	43.62	49,769.92	55.12	3,742.48	27.32	103.79	0.54
	Low	7,568.04	6.15	7,501.10	8.31	51.64	0.38	–	–
	Unsuitable	184.07	0.15	183.69	0.20	–	–	–	–

## 4 DISCUSSION

We used the niche model to evaluate the suitable habitat distribution of three raptors in the Sanjiangyuan National Park, and the results clearly showed three conclusions: (a) The suitable habitat (i.e., the total area of high and medium habitats) of Upland Buzzard and Himalayan Vulture was extremely high, accounted for more than 90% of the total area. In comparison, the suitable habitat of Saker falcon was relatively low, but more than 60%. This shows that the three species have high adaptability to the topography and climate in the Sanjiangyuan National Park. Although there was an anthropogenic influence variable, it seems that this predictor has no significant impact on the suitable habitat distribution of the three species. (b) The suitable habitat of the three species overlap greatly, and the overlap area reached 60.47% of the total area. The reason is that the habitat conditions of the three species are very similar, and the species living in the similar environment for a long time will have similar adaptability to the environment. (c) For the three species, the elevation and predictors related to temperature were the main factors affecting their habitat suitability. Especially, elevation was the most important environmental predictor. Elevation may not directly affect the distribution of the three species, but it can largely determine the food resources. Food supply has the most direct impact on animal survival, reproductive success, and population size (Newton, 2003a). In the seas of Alaska, the reproductive success of kittiwakes is dependent upon the availability, quantity, and quality of their food (Jodice et al., 2006). Raptors are limited by their food, which have been proved by overwhelming empirical evidence (Newton, 2003a; Southern, 1970). The reproductive success rate of Accipiter Gentilis declined during a period of simultaneous decline of major prey species (Rutz & Bijlsma, 2006). Sanjiangyuan National Park is located in the Qinghai–Tibet Plateau, where can provide abundant plateau food resources for the three raptors. Plateau pika, root voles, and some small finches are the main food sources of Upland Buzzard and Saker falcon (Li, Yi, Li, & Zhang, 2004; Watson & Clarke, 2000). A large number of grazed livestock carcasses and traditional celestial burials on the plateau also can provide abundant food for Himalayan Vulture (Ma & Xu, 2015). Even in winter when food is scarce, plateau pika equipped with high reproductive rate and nondormant characteristics, and a large number of livestock carcasses can still provide food for these three raptors. In addition, the preference of raptors for high elevation areas may be related to lower human disturbance (Dixon, Li, Rahman, Batbayar, & Zhan, 2017).

Temperature‐related environmental variables were another important predictor besides elevation. Temperature can impact animal reproduction and growth directly, and it can also indirectly affect animal survival by affecting the food chain. Mean diurnal range was a common environmental predictor affecting the three species. The value of this predictor can well reflect the climatic characteristics of a region. If the mean diurnal range is relatively large, it means that there is relatively high temperature and relatively strong light during the day in geography. The higher overall temperatures allow endotherms to divert metabolic energy that might otherwise be spent on maintaining body temperature into growth and reproduction (Root, 1988; Wright, Currie, & Maurer, 1993). Stronger sunlight is conducive to plant photosynthesis, which affects plant production to affect phytophagous prey, thereby affecting the food supply of raptors (Hawkins et al., 2003). The Saker falcon was also affected by the highest temperature in December and the lowest temperature in January, meaning that the species is sensitive to extreme temperatures. Studies have shown that extreme precipitation generally threatens the survival of birds (Fisher et al., 2015; Robinson, Franke, & Derocher, 2017), but the three species of Raptors are located in areas where precipitation is small and there is almost no extreme precipitation, so the impact on the three species is not significant. The importance of these two environmental variables also confirms our hypothesis. Our results also show that the human impact factor is not an important factor affecting the suitable habitat distribution of the three species, which is closely related to the low population density and the small number of tourists in the source area of the park.

Additionally, interactions between raptors, such as possible prey competition, may affect the distribution of suitable habitats. Upland Buzzard and Saker falcon have been verified to feed on live animals, and they have some similarities in food choices (Cui, Su, & Jiang, 2008; Smith & Foggin, 1999). Interspecific competition occurs when the same species consumes or occupies the common limited resources necessary for its survival or reproduction (Friedemann et al., 2016; Ricklefs & Schluter, 1993). Consequently, Upland Buzzard and Saker falcon may avoid possible food competition and separate their spatial distribution, which results in less overlap of suitable habitats. On the contrary, there is no interaction of food resources between Upland Buzzard and Himalayan Vulture because their different diets (Lu et al., 2009), so the overlapping range of their suitable habitats may be wider. However, whether the coincidence of suitable habitats of three species is related to their diet is further to be verified.

According to the distribution map of suitable habitats of the three species assessed by niche model, it can be seen that the proportion of suitable habitats of Saker falcon is the lowest among the three species, but the proportion of unsuitable habitats is 39.4%, which means that falcons should be paid more attention. On the contrary, the suitability ratios Upland Buzzard and Himalayan Vulture are very high in the Sanjiangyuan area, which means that these two species have stronger adaptability than falcons in this area. We can also clearly find that the low and unsuitable habitats of the three species are mainly concentrated in the northwest and central parts of the Yangtze River source. This is probably because annual average temperature and monthly average temperature of the Yangtze River source are the lowest in the three regions (Xu, Zhang, Lin, Xu, & Xiao, 2007), so we can consider that the three species generally do not prefer a low temperature environment and Saker falcon is most sensitive to low temperatures compared to the other two species. Although Upland Buzzard and Himalayan Vulture have wide applicability to the climatic and topographical factors of the Sanjiangyuan National Park, and the human factors related to population density, land transformation, accessibility, and electric power infrastructure have little effect on the three species (Sanderson et al., 2002), this does not completely represent that the two species are not threatened in the area. According to some studies, China invested a large amount of money to kill rodents in 2014 in order to protect the ecosystem and biodiversity of the Sanjiangyuan region, with the main target of highly fertile plateau pika (Wu & Wang, 2017). However, this method would affect the food chain of the plateau ecosystem and endanger the local biodiversity. The reason is that these poisoned animals will be captured by Upland Buzzard and Saker falcon, resulting in secondary poisoning of both species. On the contrary, livestock raised on the plateau are not exposed to toxic substances, so Himalayan Vulture may not pose a potential threat in this regard (Lu et al., 2009). In addition, it should be noted that HHI only provides human impacts on ecosystems over a period of time, while the situation beyond that period may not be accurately represented. Therefore, in order for the three species to survive better in the region, these direct or indirect threats should be taken seriously and avoided as far as possible.

In our study, maximum entropy model was used to evaluate the habitat adaptability of three species in the Sanjiangyuan National Park. Although the environmental variables used in the habitat assessment of three species were limited to climate, terrain, and human impact, they did play an important role in determining species distribution and habitat suitability based on the high precision of the selected mode. Therefore, our assessment results still have reference significance for the conservation measures and the management mechanism of the three species in this area. Based on our prediction results and analysis, we put forward two suggestions:

- We need to pay more attention to the suitable areas of the three species, especially the overlapping parts of the three species suitable areas, which should be the important areas for protection.
- Avoiding the use of toxic substances in suitable areas of the species as far as possible also recommended that a large number of electric power infrastructures should not be built in the park.

## 5 CONCLUSION

We used the maximum entropy model and three sets of environmental variables to evaluate the suitable habitat distribution of Upland buzzard, Saker falcon, and Himalayan vulture. Although the proportion of suitable habitats of the three species was more than 60% of the total park area, there were still some unfavorable factors that threaten habitat suitability. Therefore, we propose to protect the three raptors in the park by eliminating unfavorable factors and giving priority to protecting their suitable areas. At the same time, while we pay attention to and protect these three species, it is also conducive to the better survival and development of other species in the region.

## CONFLICT OF INTEREST

None declared.

## AUTHOR CONTRIBUTIONS

Tongzuo Zhang developed concept and led manuscript production. Jingjie Zhang performed the structure of manuscript and drafted the first version of the manuscript. Feng Jiang led spatial modeling and contributed to manuscript writing. Wen Qin, Hongmei Gao, Guangying Li, Shengqing Li, and Zhenyuan Cai performed the data collection and provided logistical support. Gonghua Lin contributed to data analysis. All coauthors participated in the scientific discussions and commented on the manuscript.

## DATA AVAILABILITY

We used open‐access data from GBIF (https://www.gbif.org/;), WorldClim (http://www.worldclim.org/; https://doi.org/10.1002/ece3.3994), ASTER GDEM V2 (http://www.gscloud.cn/; https://doi.org/10.7717/peerj.3477) and Last of the Wild, V2 (http://sedac.cie.columbia.edu/; https://doi.org/10.7717/peerj.3477).
