# Supplementary material for: Maxent modeling for predicting the spatial distribution of three raptors in the Sanjiangyuan National Park, China
Source: Ecol Evol. 2019 May 20;9(11):6643–54. doi: 10.1002/ece3.5243 (PMC6580265; doi:10.1002/ece3.5243)
Supplement: Supplementary file 1 [file ECE3-9-6643-s001.docx]

**Table 1**．Correlation matrix of variables selected for three species distribution model analysis. Variables are listed along the top and left side, with ρ-correlations for each variable pair given in the table. (a) Upland buzzard, (b) Saker falcon, (c) Himalayan vulture.

(a)

|  | Alt | Bio2 | Bio3 | Bio7 | Bio13 | Bio15 | Prec1 | Prec4 | Prec7 | Prec10 | HHI |
| --- | --- | --- | --- | --- | --- | --- | --- | --- | --- | --- | --- |
| Alt^[[1]](#footnote-1)^ | 1.000 |  |  |  |  |  |  |  |  |  |  |
| Bio2^[[2]](#footnote-2)^ | .545 | 1.000 |  |  |  |  |  |  |  |  |  |
| Bio3^[[3]](#footnote-3)^ | .625 | .499 | 1.000 |  |  |  |  |  |  |  |  |
| Bio7^[[4]](#footnote-4)^ | -.336 | .246 | -.684 | 1.000 |  |  |  |  |  |  |  |
| Bio13^[[5]](#footnote-5)^ | -.433 | -.500 | .144 | -.458 | 1.000 |  |  |  |  |  |  |
| Bio15^[[6]](#footnote-6)^ | .179 | .228 | .033 | .165 | -.100 | 1.000 |  |  |  |  |  |
| Prec1^[[7]](#footnote-7)^ | -.239 | -.513 | .036 | -.419 | .503 | -.441 | 1.000 |  |  |  |  |
| Prec4^[[8]](#footnote-8)^ | -.465 | -.479 | .081 | -.439 | .724 | -.561 | .666 | 1.000 |  |  |  |
| Prec7^[[9]](#footnote-9)^ | -.584 | -.605 | -.078 | -.318 | .781 | -.503 | .514 | .799 | 1.000 |  |  |
| Prec10^[[10]](#footnote-10)^ | -.313 | -.217 | .449 | -.655 | .646 | -.223 | .376 | .672 | .582 | 1.000 |  |
| HHI^[[11]](#footnote-11)^ | -.671 | -.446 | -.243 | -.070 | .468 | -.186 | .342 | .491 | .529 | .535 | 1.000 |

(b)

|  | Alt | Bio2 | Bio3 | Bio7 | Bio13 | Bio15 | Prec5 | Tmax1 | Tmean10 | Tmin12 | HII |
| --- | --- | --- | --- | --- | --- | --- | --- | --- | --- | --- | --- |
| Alt | 1.000 |  |  |  |  |  |  |  |  |  |  |
| Bio2 | .463 | 1.000 |  |  |  |  |  |  |  |  |  |
| Bio3 | .747 | .776 | 1.000 |  |  |  |  |  |  |  |  |
| Bio7 | -.712 | -.186 | -.736 | 1.000 |  |  |  |  |  |  |  |
| Bio13 | .363 | -.046 | .471 | -.786 | 1.000 |  |  |  |  |  |  |
| Bio15 | .641 | .621 | .645 | -.304 | .033 | 1.000 |  |  |  |  |  |
| Prec5^[[12]](#footnote-12)^ | -.172 | .319 | .425 | -.336 | .520 | -.141 | 1.000 |  |  |  |  |
| Tmax1^[[13]](#footnote-13)^ | -.720 | -.268 | -.262 | .131 | .245 | -.655 | .743 | 1.000 |  |  |  |
| Tmean10^[[14]](#footnote-14)^ | -.581 | -.133 | -.074 | .059 | .249 | -.158 | .695 | .772 | 1.000 |  |  |
| Tmin12^[[15]](#footnote-15)^ | -.489 | -.476 | -.375 | .054 | .107 | -.671 | .286 | .600 | .304 | 1.000 |  |
| HII | -.489 | -.476 | -.375 | .054 | .107 | -.671 | .584 | .286 | .600 | .304 | 1.000 |

(c)

|  | Alt | Bio2 | Bio3 | Bio6 | Bio7 | Bio12 | Bio14 | Bio15 | Prec1 | HII |
| --- | --- | --- | --- | --- | --- | --- | --- | --- | --- | --- |
| Alt | 1.000 |  |  |  |  |  |  |  |  |  |
| Bio2 | .266 | 1.000 |  |  |  |  |  |  |  |  |
| Bio3 | .268 | .436 | 1.000 |  |  |  |  |  |  |  |
| Bio6^[[16]](#footnote-16)^ | -.522 | -.122 | .540 | 1.000 |  |  |  |  |  |  |
| Bio7 | -.138 | .310 | -.705 | -.627 | 1.000 |  |  |  |  |  |
| Bio12 | -.238 | -.142 | .584 | .662 | -.731 | 1.000 |  |  |  |  |
| Bio14 | -.300 | -.451 | .175 | .452 | -.504 | .723 | 1.000 |  |  |  |
| Bio15 | .655 | .296 | .251 | -.284 | -.078 | -.414 | -.609 | 1.000 |  |  |
| Prec1 | -.177 | -.280 | .146 | .336 | -.349 | .497 | .723 | -.532 | 1.000 |  |
| HII | -.581 | -.155 | .078 | .569 | -.180 | .384 | .196 | -.328 | .172 | 1.000 |

**Table 2**．Frequency distribution of species environment variables with contribution rate above 10% . A, Upland buzzard: (a) Altitude (m), (b) Mean Diurnal Range (°C); B, Saker falcon: (a) Altitude (m), (b) Mean Diurnal Range (°C), (c) Maximum Temperature Range(°C), (d) Minimum Temperature Range(°C); C, Himalayan vulture: (a) Altitude (m), (b) Isothermality.

A

*,
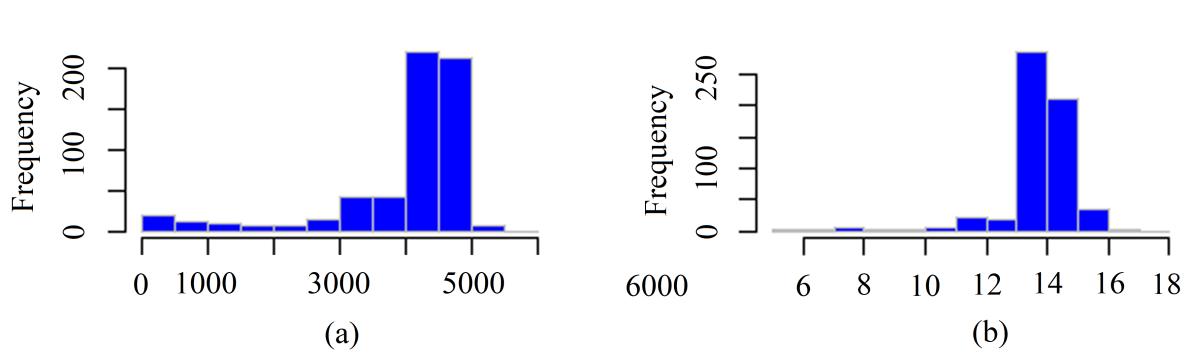
*

B


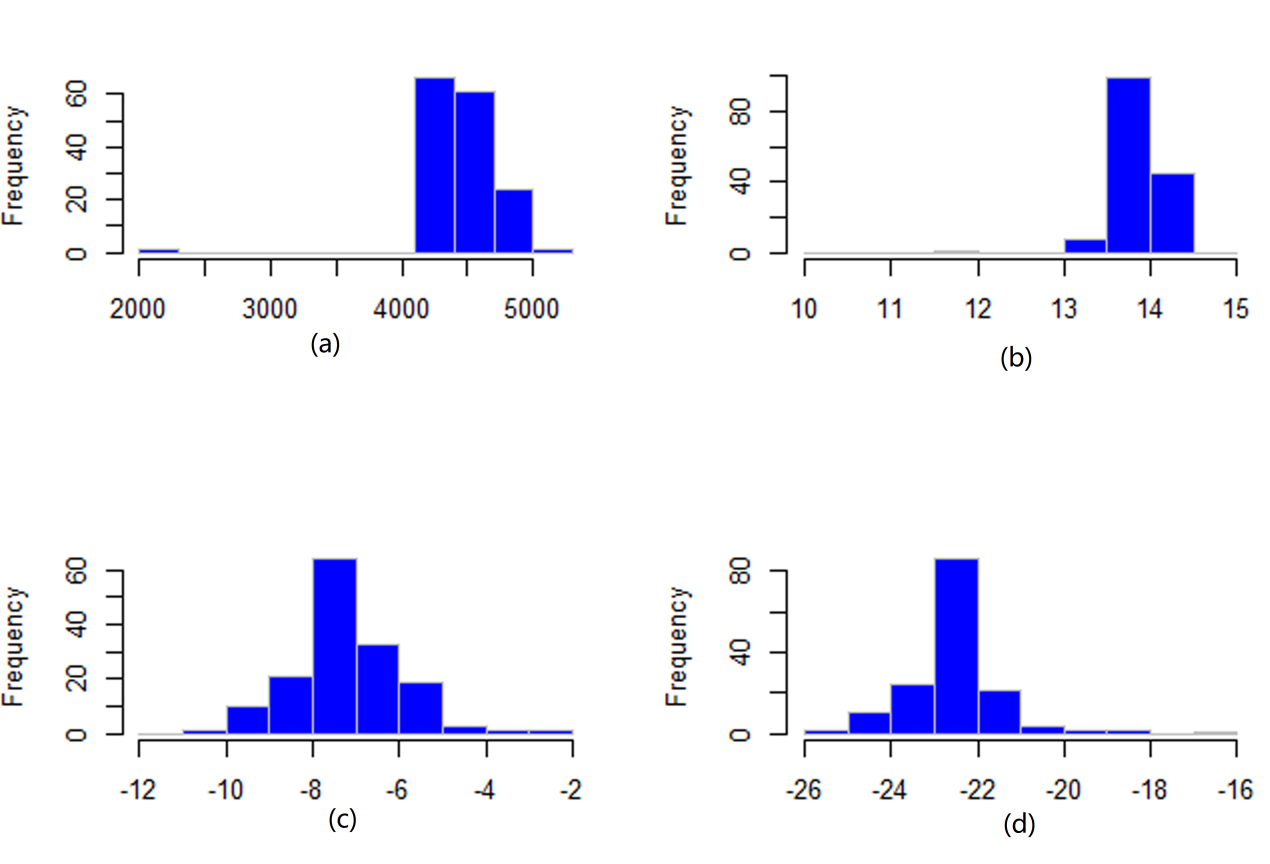


C


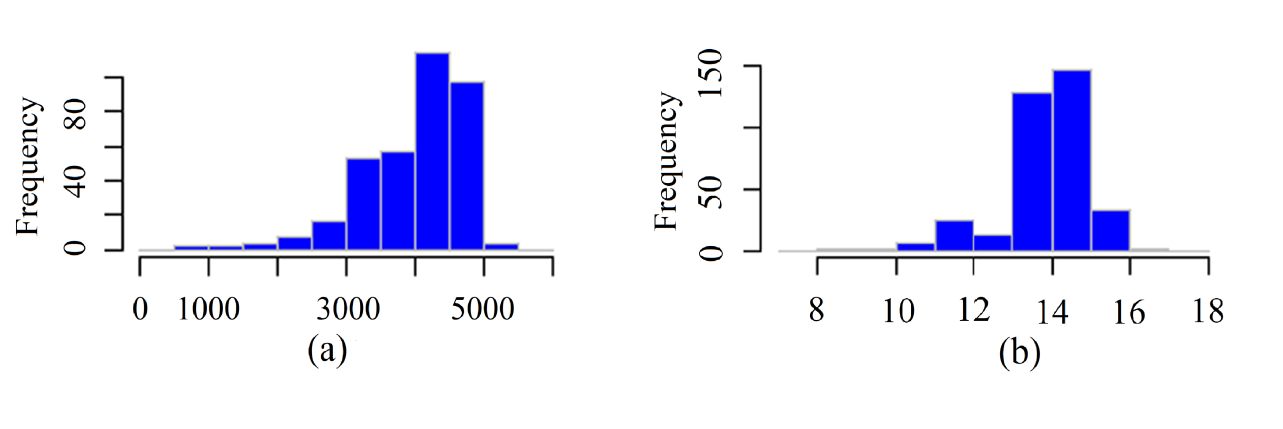


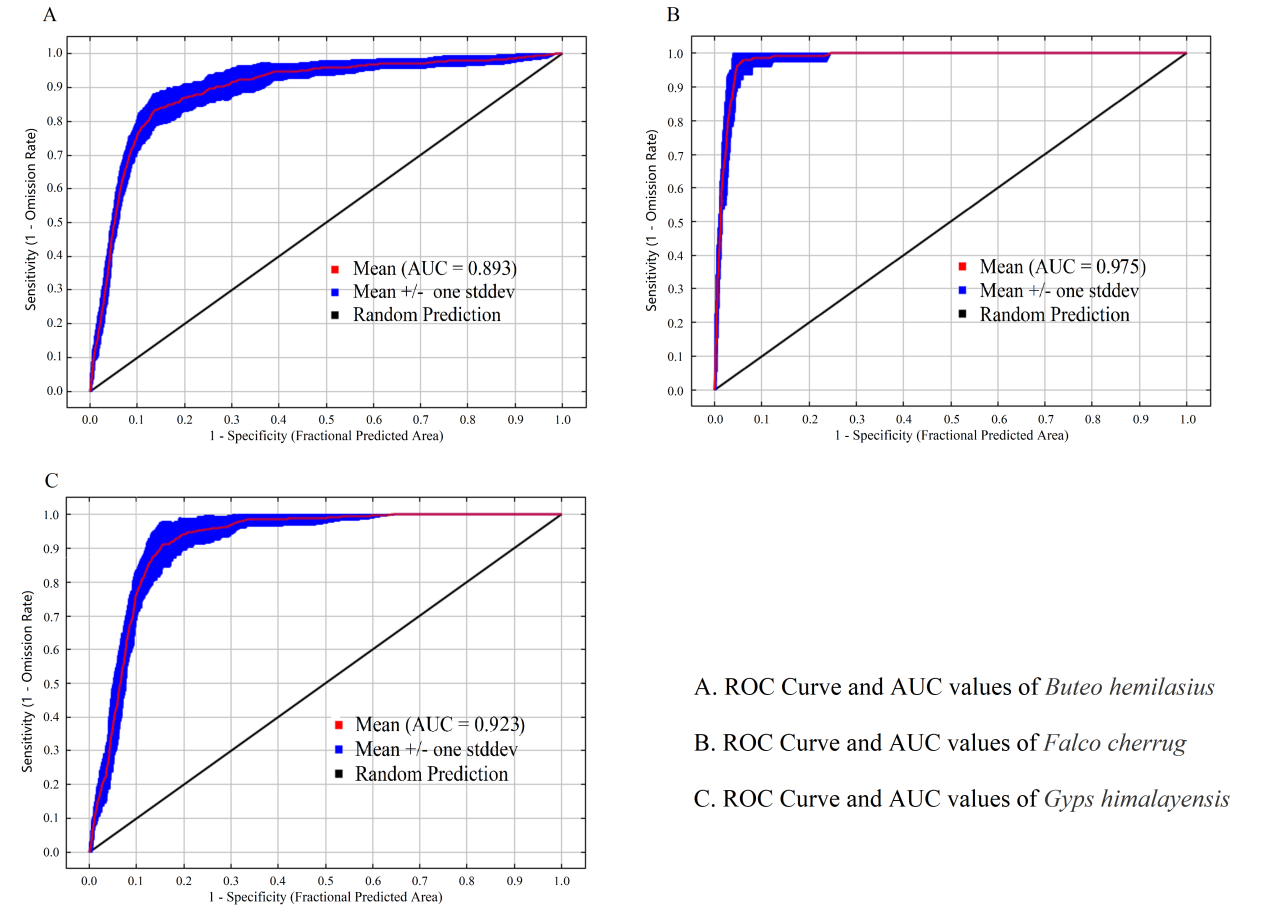


**Figure 1** Results of the Model performance; ROC Curves and AUC values of the three species. A, Upland Buzzard; B, Saker falcon ; C, Himalayan Vulture.


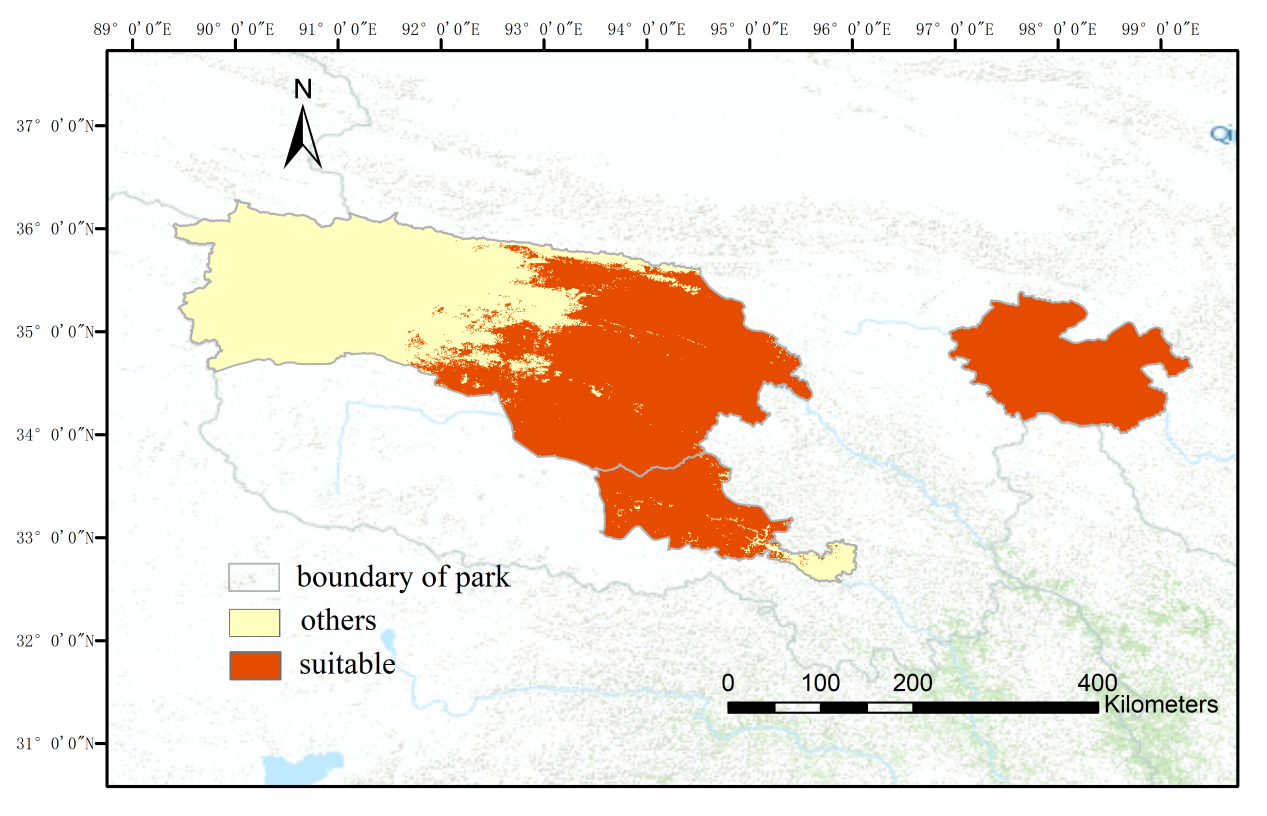


**Figure 2** Suitable Habitat Distribution Map of Three Species Overlapping

1. Elevation [↑](#footnote-ref-1)
2. Mean Diurnal Range [↑](#footnote-ref-2)
3. Isothermality (BIO2/BIO7)(*100) [↑](#footnote-ref-3)
4. Temperature Annual Range [↑](#footnote-ref-4)
5. Precipitation of Wettest Month [↑](#footnote-ref-5)
6. Precipitation Seasonality [↑](#footnote-ref-6)
7. January precipitation [↑](#footnote-ref-7)
8. April precipitation [↑](#footnote-ref-8)
9. July precipitation [↑](#footnote-ref-9)
10. October precipitation [↑](#footnote-ref-10)
11. Human Influence Index [↑](#footnote-ref-11)
12. May precipitation [↑](#footnote-ref-12)
13. January maximum temperature [↑](#footnote-ref-13)
14. October mean temperature [↑](#footnote-ref-14)
15. December minimum temperature [↑](#footnote-ref-15)
16. Min Temperature of Coldest Month [↑](#footnote-ref-16)
